# Optimizing Kidney Allograft Years: Underutilization of Pediatric Donors

**DOI:** 10.1097/TXD.0000000000001891

**Published:** 2025-12-12

**Authors:** Nicholas S. Herrera, Ana Cortez, Diane M. Cibrik, Bradley A. Warady

**Affiliations:** 1 Division of Nephrology, Department of Internal Medicine, University of Kansas Medical Center, Kansas City, KS.; 2 Division of Nephrology, Department of Pediatrics, Children’s Mercy Kansas City, Kansas City, MO.

## Abstract

**Background.:**

The Kidney Donor Profile Index (KDPI) score drives kidney allocation in the United States for both adult and pediatric donors, despite the calculation being primarily derived from adult donor data. A KDPI of ≤20% is considered to have the longest graft survival potential and is typically prioritized for recipients who have a low Estimated Posttransplant Survival (EPTS) score. Using KDPI to assess pediatric donor quality may underestimate graft survival and result in underutilization of these kidneys for recipients with a low EPTS score.

**Methods.:**

Using Scientific Registry of Transplant Recipients data, we analyzed 5-y posttransplant death-censored graft survival of kidneys from donors aged 0–<4, 4–<8, 8–<12, and 12–<18 y compared with adult donor kidneys with a KDPI of ≤20% to adult recipients using Kaplan-Meier analysis, pairwise comparisons, and multivariate Cox proportional models.

**Results.:**

Death-censored graft survival was not significantly different for pediatric donors aged 8–<12 y (χ^2^ = 0.8, *P* = 0.37) and 12–<18 y (χ^2^ = 0.23, *P* = 0.63) when compared with adult donor kidneys. Five-year graft survival was 91.8%, 92.3%, and 92.4%, respectively, with a mean KDPI of 37.5% (range, 1%–85%), 16.2% (range, 0%–85%), and 10.7% (range, 0%–19%) and mean EPTS score of 42.9%, 29.9%, and 22.8%, respectively.

**Conclusions.:**

Graft survival for kidneys from donors aged 8–18 y is not inferior to that of adult donors with a KDPI of ≤20%. These data support the use of pediatric donors aged 8 y and older for those recipients who traditionally received adult donor kidneys with a KDPI of ≤20%.

## INTRODUCTION

Kidney transplantation is the replacement therapy of choice for people with kidney failure. In 2024, in the United States, 27 759 kidney alone transplants were performed on the basis of Organ Procurement and Transplantation Network (OPTN) data as of January 31, 2025. Nearly 77% of adult and 70% of pediatric recipients received a kidney from a deceased donor.^1^ Although pediatric donor kidneys are used in kidney transplantation, the majority are allocated to adult recipients and in some cases, to adult recipients older than 54 y.^2^

Historically, placement of small kidneys into infants, young children, and adults was met with inferior graft survival.^3-6^ More than 30 y ago, Harmon et al^3^ reported technical complications and thrombosis among the main reasons for poor outcomes in recipients of pediatric allografts. However, these issues were primarily due to the utilization of pediatric donors younger than 6 y. In fact, very young pediatric donors younger than 2 y had graft survival rates markedly inferior to those of older pediatric donor kidneys.^7^ Concerns about small donor size have influenced allocation and acceptance practices so much that the use of deceased donor transplants from donors younger than 10 y declined substantially from 35% in 1987 to just 5% in 2010.^8^

Given the shortage of donor organs, a primary goal of transplantation is to maximize graft survival. A new Kidney Allocation System (KAS) was implemented in 2014 to reduce discard rates and to optimize graft survival.^9^ To accomplish this, a Kidney Donor Risk Index (KDRI) model using Scientific Registry of Transplant Recipients (SRTR) data was created that incorporated 10 predictive donor factors that influenced graft survival. However, because the KDRI score is primarily based on adult donor data and outcomes, it may not be applicable to pediatric donors. The Cox regression model produces KDRI scores that diverge at 18 y of age, after which donors younger than 18 y are associated with higher KDRI scores.^10,11^ Young donors have higher KDRI scores because the Cox model incorporates mathematical restrictions such as age younger than 18 y, height <170 cm, and weight <80 kg. Other markers, such as hypertension and diabetes, also influence KDRI but are less relevant in the pediatric population. This has led to criticism of using KDRI (later known as KD Profile Index [KDPI]) in pediatric donors to assess kidney quality and predict graft survival, as the scores vary greatly and are primarily driven by age, height, and weight.^11^

One of the goals of the new KAS system is to prioritize allografts with the lowest KDPI to recipients with the longest expected survival. Currently, pediatric recipients are given priority for allografts with a KDPI of <35%. Despite the motivation behind the United Network for Organ Sharing policy to prioritize pediatric recipients to receive those allografts with the best overall graft survival, the OPTN 2022 Annual Data Report showed that approximately 3% of pediatric recipients receive a kidney transplant with a KDPI of >35%.^1^

In addition, the Estimated Posttransplant Survival (EPTS) score was created to assess adult recipient life expectancy to optimize kidney donor allocation; a recipient with an EPTS score of ≤20% is typically prioritized to receive a kidney donor with a KDPI ≤20%. Similarly, the EPTS score was developed using only OPTN adult data. Although the EPTS score is low for most pediatric patients, the EPTS score is less reliable for predicting pediatric posttransplant survival outcomes and is not used for donor allocation in pediatrics.^12^

To assess the widely held belief that kidneys from pediatric donors are suboptimal when compared with adult donor kidneys, we addressed 3 questions using SRTR data: (1) do pediatric donor kidneys perform as well as kidneys from an adult donors with a KDPI of ≤20% and if so, at what age; (2) are pediatric donor kidneys being placed into recipients who will have the maximum life year benefit from a kidney transplant^13,14^; and (3) are pediatric donor kidneys underused in pediatric recipients. Our hypothesis is that kidneys from pediatric donors within a certain age have graft outcomes equal to or better than kidneys from a deceased donor with a KDPI of ≤20% and would be an acceptable alternative for any pediatric or young adult recipient in need of a kidney transplant.

## MATERIALS AND METHODS

We performed a retrospective study using the SRTR database to assess pediatric donor kidney outcomes compared with adult kidney donors with a KDPI of ≤20% (“ideal deceased donor”). Our main analysis pertains to adult recipients, defined as 18 y old and older, who received a deceased donor kidney-alone transplant from January 1, 2014, to October 1, 2023. The start date marks the initial implementation and recording of KDPI by OPTN for donor allocation. For this analysis, recipients were excluded if they were younger than 18 y, had a prior transplant or multiple allografts, had a living or pediatric en bloc donor, or received a kidney from an adult donor with a KDPI of >20%. Only adults who received a kidney donor with a documented KDPI at the time of transplant were included in the analysis. We chose to exclude pediatric recipients from the main analysis because the majority of pediatric donor kidneys are allocated to adult recipients and different causes of graft loss.

Pediatric donors were segregated regardless of their reported KDPI at the time of donation and grouped by age for easier comparison with the ideal adult deceased donor. Initial group comparisons compared pediatric donors at each year of life using Kaplan-Meier plots for death-censored graft survival (data not shown). Donor ages that performed similarly were grouped together using the pediatric donor survival curves for each year of life. The final comparison groups were pediatric donors aged 0–<4, 4–<8, 8–<12, and 12–<18 y and adult deceased donors with a KDPI of ≤20%. Pairwise comparison was used for each pediatric group to determine significance between groups.

For the main analysis, we analyzed death-censored graft survival of pediatric donor kidneys in adult recipients using univariate Kaplan-Meier analysis and pairwise comparisons with log-rank tests, as well as multivariate Cox proportional hazard models at 5 y posttransplant. We chose not to pursue a competing risk model for predicting allograft failure because such models are more applicable to groups with a higher likelihood of patient death, such as older recipients, who are not included in this analysis.^15^ All variables listed in the donor and recipient demographic tables were considered for inclusion in the multivariate model. Given the relatively low number of pediatric donor transplants, the lower number of graft failures in the youngest donor groups, and similar recipient demographics, we chose to limit the variables to a best-fitting model to avoid overfitting. We used forward, backward, and stepwise regression techniques to choose the best model using the Akaike information criterion and the Bayesian information criterion. Among similarly performing models, we selected the model that included the most important clinical factors influencing allograft failure based on our experience. The final covariates included in the Cox proportion hazards model were donor groups, delayed graft function, ESKD etiology, 0 HLA mismatch, and recipient sex and race. The large sample size of the data set and robust plots of the Schoenfeld residuals support no practical importance of deviations from the proportion hazard assumption. However, to alleviate concern for relevance, the sandwich estimator was used to address any heteroscedasticity among observations.^16,17^ The Efron method was used for tied values in the Cox models.

The EPTS score was retrospectively calculated for each transplant recipient at the time of transplant using the OPTN-guided equation to derive the EPTS raw value, then converting it to the familiar EPTS score.^18^ The EPTS scores were validated with 100 randomly selected recipients at our institution.

To address aim 3, descriptive statistics of pediatric recipients were conducted using the same transplant timeframe noted in the main analysis. This includes the total number of pediatric recipients who received a kidney alone from a pediatric donor or an adult donor with a KDPI of >20%. The totals were further divided by KDPI as follows: 20%–<30%, 30%–<40%, 40%–<50%, and ≥50%.

The data reported here were supplied by the Hennepin Healthcare Research Institute, the contractor for the SRTR. The interpretation and reporting of these data are the responsibility of the authors and in no way should be seen as an official policy of or interpretation by the SRTR or the US Government. This study used data from the SRTR. The SRTR data system includes data on all donor, waitlisted candidates, and transplant recipients in the United States, submitted by the members of the OPTN. The Health Resources and Services Administration and the US Department of Health and Human Services provides oversight to the activities of the OPTN and SRTR contractors. This was a retrospective analysis of a large de-identified database without external data linkage; therefore, IRB approval was not required. All data processing and analyses were conducted using R statistical software (version 4.4.1, R Core Team, 2024. R: A Language and Environment for Statistical Computing. R Foundation for Statistical Computing, Vienna, Austria. http://www.r-project.org).

## RESULTS

**FIGURE 1.**
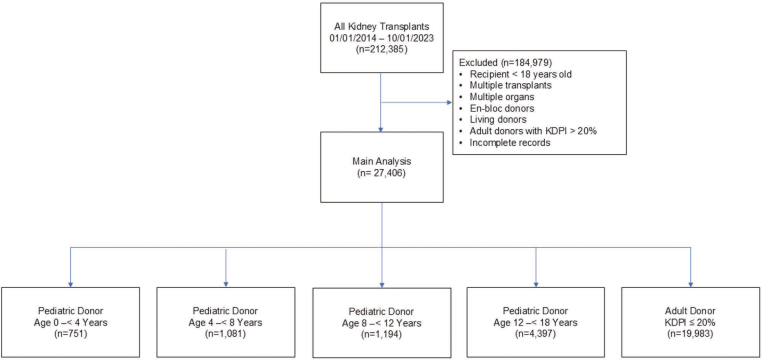
Cohort diagram demonstrating the study population. KDPI, Kidney Donor Profile Index.

Figure 1 displays the final 5 cohorts used in the analysis which consisted of pediatric donors aged 0–<4, 4–<8, 8–<12, and 12–<18 y and adult deceased donors with a KDPI of ≤20%; there were 27 406 kidney transplants in our analysis overall with 751, 1081, 1194, 4397, and 19 983 kidney transplants in each cohort, respectively, and a total of 7423 pediatric donor kidneys.

**FIGURE 2.**
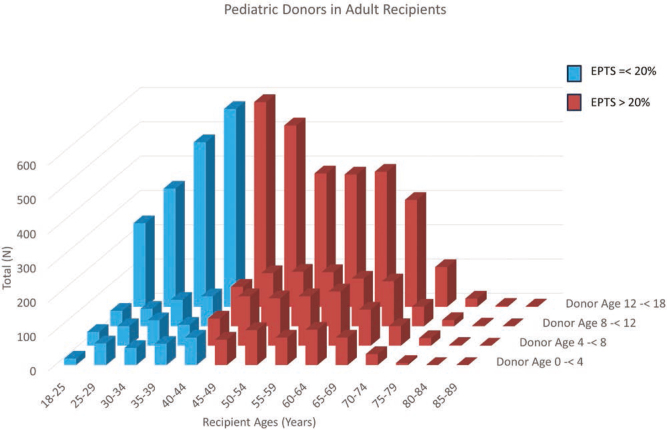
Three-dimensional histogram of pediatric donors and recipients by age. EPTS, Estimated Posttransplant Survival.

Figure 2 demonstrates that donor age is associated with a higher KDPI with an inflection point at 18 y of age. Pediatric donors younger than 8 y are likely to have a KDPI >35%.

Donor demographics are depicted in Table 1. Approximately 89.9% of pediatric donor kidneys (N = 7423) were allocated to adult recipients, whereas the remaining 10.1% (N = 834) were allocated to pediatric recipients. Donor demographics were different among the cohorts as anticipated, given the design of the analysis. Donor race was the most significant difference between pediatric and adult donors, with a greater percentage of adult donors being White (92.2%). In contrast, there was a higher proportion of Black donors among pediatric donors. Donors after circulatory death (DCD) rates were lower for all groups compared with the national cohort (26.3%).^1^ In pediatric donors aged 0–<4 y and aged 4–<8 y and adult donors, the lowest percentage of DCD kidneys was 16.1%, 16.1%, and 16.0%, respectively. The pediatric donor KDPIs consisted of a wide range of values. Donors aged 0–<4 y had KDPIs ranging from 40% to 97%, whereas pediatric donors aged 12–<18 y had KDPIs ranging from 0% to 85%.

**TABLE 1.** Donor demographics: pediatric donors and adult donors with KDPI ≤20%

Demographics	Age 0–<4 y	Age 4–<8 y	Age 8–<12 y	Age 12–<18 y	KDPI ≤20%
Frequency	751 (2.7%)	1081 (3.9%)	1194 (4.4%)	4397 (16.0%)	19 983 (72.9%)
Mean donor age, y	2.5 (±1.0)	6.0 (±1.2)	10.2 (±1.2)	15.6 (±1.6)	26.4 (±5.9)
Mean donor height, cm	90.9 (±12.3)	116.0 (±11.2)	140.0 (±13.1)	168.0 (±13.0)	176.0 (±9.24)
Mean donor weight, kg	14.8 (±4.50)	24.0 (±7.00)	40.0 (±14.8)	67.8 (±19.5)	85.4 (±20.6)
Donor race White Asian Black Multi Native Pacific	78.2% 3.1% 16.4% 1.6% 0.4% 0.4%	69.9% 2.4% 25.4% 0.9% 1.0% 0.3%	69.4% 2.6% 26.0% 0.8% 0.4% 0.7%	78.7% 1.8% 17.9% 0.5% 0.8% 0.2%	92.2% 1.6% 4.2% 0.5% 1.0% 0.5%
DCD yes	16.1%	16.1%	23.9%	23.7%	16.0%
Mean KDPI	65.4% (±11.4)	51.9% (±13.5)	37.5% (±15.8)	16.2% (±13.4)	10.7% (±5.4)
KDPI Minimum First quartile Median Third quartile Maximum	40% 57% 64% 73% 97%	17% 43% 50% 60% 91%	1% 26% 37% 48% 85%	0% 6% 13% 24% 85%	0% 6% 11% 15% 19%

DCD, donor after circulatory death; KDPI, Kidney Donor Profile Index.

**FIGURE 3.**
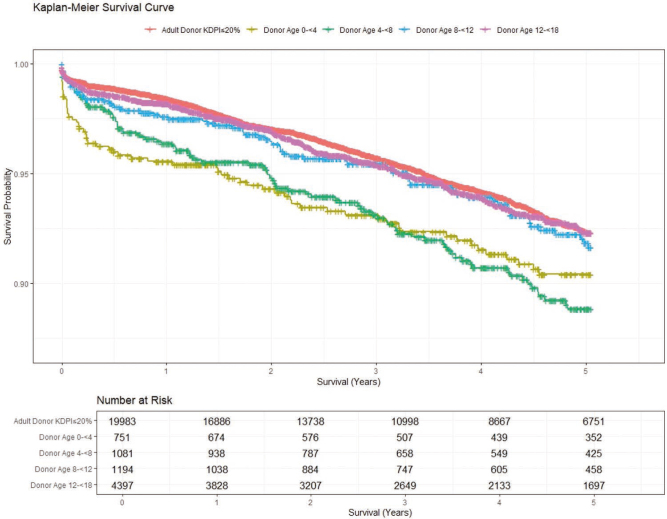
Kaplan-Meier plot of death-censored graft survival for pediatric donors and adult donors with KDPI <20%. KDPI, Kidney Donor Profile Index.

Adult recipient demographics are depicted in Table 2. The national mean age for all adult recipients at the time of transplant who received a deceased donor kidney was 54.6 y during the study period; the mean recipient age across the 5 donor groups was below the national average, ranging from 43 to 51 y. The youngest recipients were those who received kidneys from pediatric donors aged 12–<18 y and from adult donors with a KDPI of ≤20%, with a mean age of 45.8 and 43.1 y, respectively. The average EPTS score of adult recipients who received a pediatric kidney donor was higher (29.9%–44.4%) compared with those who received an adult kidney donor with a KDPI ≤20% (22.8%). Approximately 53.5% (N = 3971) of all pediatric donor kidneys are allocated to adult recipients with an EPTS score >20% as seen in Figure 3 (red bars).

**TABLE 2.** Recipient demographics: pediatric donors and adult donors with KDPI ≤20%

	Age 0–<4 y	Age 4–<8 y	Age 8–<12 y	Age 12–<18 y	KDPI ≤20%
Frequency	751 (2.7%)	1081 (3.9%)	1194 (4.4%)	4397 (16.0%)	19 983 (72.9%)
Mean recipient age, y	50.1 (±13.8)	51.6 (±13.8)	51.1 (±13.5)	45.8 (±13.8)	43.1 (±12.8)
Mean recipient height, cm	165.0 (±10.1)	166.0 (±11.8)	167.0 (±11.3)	169.0 (±11.9)	170.0 (±11.7)
Mean recipient weight, kg	67.8 (±14.8)	72.9 (±16.5)	76.6 (±17.9)	81.4 (±20.6)	82.2 (±20.7)
Mean HLA mismatch	4.34 (±1.40)	4.26 (±1.46)	4.21 (±1.42)	4.24 (±1.40)	4.24 (±1.34)
Mean EPTS	39.5 (±23.3)	44.4 (±29.9)	42.9 (±29.4)	29.9 (±27.7)	22.8 (±23.3)
Mean cPRA	16.8 (±29.7)	25.8 (±36.0)	26.6 (±36.7)	23.4 (±35.3)	20.9 (±33.3)
HLA DR match DR 0 DR 1 DR 2	13.3% 48.5% 38.2%	16.2% 47.1% 36.7%	15.3% 47.8% 36.9%	15.0% 49.0% 36.0%	14.3% 50.1% 35.6%
0-HLA mismatch	3.9%	5.3%	4.7%	4.3%	3.1%
Female sex	51.9%	53.7%	51.8%	44.0%	39.8%
Recipient race White Asian Black Multi Native Pacific	57.0% 18.4% 23.3% 0.9% 0.4% 0%	53.4% 10.9% 33.2% 0.6% 1.4% 0.6%	54.1% 9.1% 34.3% 1.0% 1.0% 0.4%	56.9% 6.9% 33.3% 1.1% 1.1% 0.5%	58.4% 7.5% 31.6% 1.0% 0.9% 0.7%
Induction Other Alemtuzumab Basiliximab Thymoglobulin	6.7% 12.4% 12.0% 69.0%	9.5% 15.0% 15.1% 60.4%	8.7% 14.6% 16.4% 60.3%	11.2% 12.7% 18.5% 57.6%	12.3% 12.9% 18.4% 56.4%
Maintenance immunosuppression: Tacrolimus/tacrolimus XR MMF/MPA Cyclosporine	93.4% 100.0% 0.5%	94.5% 99.3% 1.3%	95.2% 99.3% 0.8%	96.2% 99.5% 1.1%	96.6% 98.0% 1.0%
ESKD: Diabetes mellitus Hypertension Familial disease Glomerulonephritis Other	23.70% 26.60% 1.60% 26.40% 13.60%	28.20% 23.20% 0.80% 23.80% 15.50%	27.00% 24.50% 2.10% 22.00% 16.20%	17.20% 25.50% 1.70% 28.90% 19.30%	11.20% 25.30% 1.70% 30.90% 22.90%
Delayed graft function	19.7%	19.2%	21.4%	20.1%	17.6%
Cold ischemic time <12 12–20 ≥20	23.3% 36.4% 40.1%	28.2% 39.4% 32.1%	28.0% 38.4% 33.3%	29.7% 36.2% 33.8%	32.1% 36.9% 30.6%

cPRA, calculated panel-reactive antibody; EPTS, Estimated Posttransplant Survival; ESKD, end-stage kidney disease; KDPI, Kidney Donor Profile Index; MMF, mycophenolate mofetil; MPA, mycophenolic acid; XR, extended release.

**FIGURE 4.**
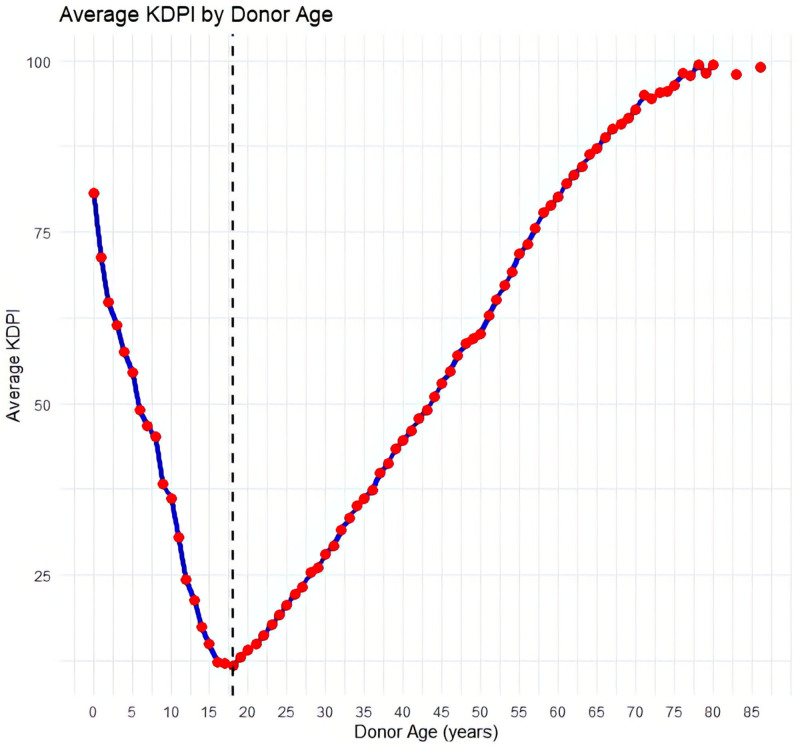
Average KDPI by donor age. KDPI, Kidney Donor Profile Index.

Figure 4 shows the Kaplan-Meier 5-y death-censored kidney graft survival from adult donors with a KDPI ≤20% versus pediatric donor cohorts. Graft survival of adult donors with a KDPI ≤20% was 92.4% and served as the reference group for the following comparisons. The Kaplan-Meier plot for death-censored graft survival was statistically worse in kidneys from pediatric donors aged 0–<4 y (log-rank, χ^2^ = 7.4, *P* = 0.006) and 4–<8 y (log-rank, χ^2^ = 17.3, *P* < 0.001), with 5-y graft survival being 90.4% and 88.8%, respectively. In contrast, the Kaplan-Meier plot for death-censored graft survival was not significantly different in the pediatric donors aged 8–<12 y (log-rank, χ^2^ = 0.8, *P* = 0.37) and 12–<18 y (log-rank, χ^2^ = 0.23, *P *= 0.63), with 5-y graft survival results being 91.8% and 92.3%, respectively. Using pairwise comparisons, pediatric donors aged 0–<4 and 4–<8 y were not statistically different from each other (log-rank, χ^2^ = 0.23, *P* = 0.63). Similarly, pairwise comparisons for pediatric donors aged 8–<12 and 12–<18 y were not statistically different (log-rank, χ^2^ = 0.32, *P* = 0.57). In the multivariate Cox model, when compared with adult donors with a KDPI ≤20%, death-censored graft loss of kidneys from pediatric donors aged 0–<4 y was 57.3% higher (hazard ratio [HR], 1.573; 95% confidence interval [CI], 1.204-2.055, *P *< 0.001; Table 3). Likewise, kidneys from pediatric donors aged 4–<8 y had a 64.4% higher risk of graft loss (HR, 1.644; 95% CI, 1.321-2.046; *P* < 0.001; Table 3). In contrast, pediatric kidney donors aged 8–<12 y did not exhibit a statistically significant difference in the risk for graft loss (HR, 1.120; 95% CI, 0.878-1.428; *P* = 0.362; Table 3) compared with adult donor kidneys with a KDPI ≤20%. Similarly, pediatric kidney donors aged 12–<18 y did not have a statistically significant difference in the risk for graft loss (HR, 1.020; 95% CI, 0.886-1.175; *P* = 0.781; Table 3).

**TABLE 3.** Multivariate Cox analysis for death-censored graft loss

	HR (95% CI)	*P*
Donor age (reference: KDPI ≤20%) 0–<4 4–<8 8–<12 12–<18	1.573 (1.204-2.055) 1.644 (1.321-2.046) 1.120 (0.878-1.428) 1.020 (0.886-1.175)	<0.001 <0.001 0.362 0.781
Delayed graft function	2.023 (1.804-2.268)	<0.001
ESKD diagnosis (reference: other) Cystic disease Diabetes mellitus Familial disease Glomerulonephritis Hypertension	0.688 (0.526-0.901) 0.802 (0.658-0.978) 0.876 (0.533-1.439) 1.297 (1.116-1.508) 1.066 (0.908-1.251)	0.006 0.028 0.601 <0.001 0.435
0 HLA mismatch	0.698 (0.490-0.995)	0.047
Recipient race (reference: White) Asian Black Multi Native Pacific	0.646 (0.501-0.833) 1.774 (1.584-1.986) 1.186 (0.673-2.089) 1.136 (0.658-1.961) 1.799 (1.028-3.147)	<0.001 <0.001 0.555 0.647 0.040
Recipient sex: male	0.892 (0.802-0.992)	0.036

CI, confidence interval; ESKD, end-stage kidney disease; HR, hazard ratio.

Finally, we examined whether pediatric donor kidneys were underused in pediatric recipients. In our data set, there were 6470 pediatric recipients who included 4418 who received a deceased donor allograft; of these, 74.1% (N = 2552) received adult donor kidneys with a KDPI ≤20% (data not shown). Table 4 shows the distribution of pediatric recipients (N = 1181) who received a kidney either from an adult (Table 4) or pediatric (Table 5) donor with KDPI >20% during the study period. Of the 3442 pediatric deceased donor recipients who received an adult donor, 25.9% (N = 890) received a kidney transplant with a KDPI of >20% (Table 4, column 2). Most of these recipients (N = 631) received an adult kidney donor with a KDPI between 20% and 30%; 259 pediatric recipients (approximately 7.5%) received an adult kidney donor with a KDPI >30%. Approximately 3.2% (N = 110) of pediatric deceased donor recipients received an adult kidney donor with a KDPI >35% (data not shown). Our data set shows that approximately 4.4% (N = 194) of all pediatric deceased donor recipients received a kidney from a donor with a KDPI >35% (data not shown). In contrast to adult donors, 976 pediatric recipients received a deceased pediatric donor kidney, of which 291, approximately 32.7%, were from a pediatric donor with a KDPI >20% (Table 5, column 2). The average ages of the pediatric donors allocated to pediatric recipients were 15.3 (±2) y and 11.4 (± 4) y for KDPI of <20% and KDPI >20%, respectively.

**TABLE 4.** Pediatric recipients with transplant from adult donors

Recipient age, y	Total with KDPI >20%	KDPI 20%–< 30%, donor age ≥18 y	KDPI 30%–< 40%, donor age ≥18 y	KDPI 40%–<50%, donor age≥18 y	KDPI ≥50%, donor age ≥18 y
0–<1	1	1	0	0	0
1–<2	13	8	3	2	0
2–<3	31	22	7	1	1
3–<4	54	44	8	1	1
4–<5	32	24	6	2	0
5–<6	32	23	7	1	1
6–<7	28	15	12	0	1
7–<8	29	19	9	0	1
8–<9	24	18	6	0	0
9–<10	33	26	7	0	0
10–<11	40	33	6	1	0
11–<12	51	37	12	2	0
12–<13	48	28	17	2	1
13–<14	67	49	18	0	0
14–<15	73	57	14	0	2
15–<16	82	56	22	3	1
16–<17	89	54	33	2	0
17–<18	163	117	40	5	1
Total	890	631	227	22	10

KDPI, Kidney Donor Profile Index.

**TABLE 5.** Pediatric recipients with transplant from pediatric donors

Recipient age, y	Total with KDPI >20%	KDPI 20%–<30%, donor age <18 y	KDPI 30%–<40%, donor age <18 y	KDPI 40%–<50%, donor age <18 y	KDPI ≥50%, donor age <18 y
0–<1	1	0	1	0	0
1–<2	5	3	2	2	1
2–<3	15	10	2	0	3
3–<4	12	8	0	1	3
4–<5	21	15	1	1	4
5–<6	10	1	5	3	1
6–<7	21	11	3	5	2
7–<8	15	9	4	1	1
8–<9	14	8	6	0	0
9–<10	15	9	5	1	0
10–<11	11	5	4	1	1
11–<12	11	3	4	3	1
12–<13	15	11	1	3	0
13–<14	22	16	5	1	0
14–<15	13	9	1	1	2
15–<16	24	13	3	4	4
16–<17	28	17	6	5	0
17–<18	35	19	8	4	4
Total	291	167	61	36	27

KDPI, Kidney Donor Profile Index.

## DISCUSSION

Using the SRTR database, our analyses have shown that pediatric donor kidneys >8 y of age perform as well in adult recipients as kidneys from adult donors with a KDPI of ≤20%. In addition, approximately 53.3% (N = 3956) of all pediatric donor kidneys are allocated to adult recipients with an EPTS score >20%. Only 22.1% of pediatric deceased donor recipients (n = 976) received a kidney transplant from a pediatric donor, mostly older than 15 y of age. Therefore, our analysis demonstrates that pediatric kidney donors are not being used to maximize the life-year benefit for recipients with the highest probability of long-term survival.

In addition, our analysis suggests that even those pediatric donors younger than 8 y have a similar 5-y death-censored graft survival as compared with the average of all adult deceased donor kidneys at approximately 89% and 90.4%, respectively, with an averaged donor KDPI of 57.4% and 48.1%, respectively (data not shown). This argues that pediatric kidney donors younger than 8 y have better death-censored graft survival than predicted by their KDPI. The sharp increase in KDPI for donors younger than 18 y appears to be a mathematical consequence driven primarily by donor age, height, and weight, all of which are correlated and based on our analysis underestimates their allograft potential. Although nephron mass is fully developed by 36 wk of gestation, donor-recipient size mismatch leading to hyperfiltration injury may explain why donors younger than 8 y have a lower 5-y death-censored graft survival.^19,20^

Despite the goal of the new KAS allocation to extend kidney allograft survival, Husain et al in 2019 suggested that roughly 28.5% of adult patients on the kidney transplant waitlist have an EPTS score <20% but only half of them received a kidney with a KDPI ≤20%.^21^ In our analysis, pediatric donors between 8 and 18 y of age (representing 75.3% of all pediatric donors) were allocated to recipients with an average EPTS of 32.6% and recipient age of 46.9 y. In contrast, adult kidney donors with a KDPI of ≤20% were allocated to recipients with a mean EPTS of 22.8%. Thus, further optimization of the allocation system for pediatric donor kidneys may benefit adult transplant recipients with an EPTS score of ≤20% maximizing their graft survival years.

Although pediatric recipients are given priority on the deceased donor waitlist, approximately 25.9% of pediatric deceased donor recipients received an adult donor kidney with a KDPI >20% and 3.2% received an adult donor kidney with a KDPI >35%. Despite pediatric kidney donors 8–<18 y old having similar death-censored graft survival to adult kidney donors with a KDPI of ≤20% for adult recipients, only 14.4% of the 5591 pediatric donors in that age range were allocated to pediatric recipients. A potential explanation for why some pediatric recipients received adult allografts could be due to a high calculated panel-reactive antibody, but the mean calculated panel-reactive antibody of pediatric recipients who received an adult kidney donor with a KDPI >20% and 35% was 10.3% and 8.7%, respectively (data not shown). Our analysis challenges the current, accepted practice among pediatric transplant centers to preferentially accept adult donor kidneys for their patients. Although a pediatric transplant team may not accept pediatric donor kidneys for their pediatric recipients based largely on historical data, our analysis suggests that this recommendation should be restricted to those donors younger than 8 y. Pediatric programs who will accept kidneys from pediatric donors aged 8–<18 y will used an important source of high-quality kidney allografts to better meet the needs of the pediatric transplant population.

Multiple well-known publications as far back as 2005 have supported the use of small pediatric kidneys or pediatric en bloc kidneys with outcomes similar to those of the “ideal deceased donor”^6,22-30^; all are published using data before the new KAS system or did not analyze outcomes according to KDPI. These publications focused on pediatric en bloc or kidneys from small pediatric donors weighing <21 kg or younger than 5 y,^22,24^ had limited follow-up or small sample sizes,^25,27^ were from a single center,^26^ and/or were from a different country/patient population.^29^ In 2019, Suneja et al^30^ using SRTR data published on single pediatric kidney outcomes and concluded that pediatric kidney donors between 5 and 30 kg had better death-censored graft survival than an expanded criteria donor kidney. Sampaio et al^6^ analyzed United Network for Organ Sharing data from 2000 to 2019, comparing pediatric donors by weight, and found that single kidneys from donors weighing 15–18 kg had 10-y graft survival rates similar to those of single kidneys from pediatric donors weighing >20 kg. Additionally, Sampaio et al reported that donors weighing between 10 and 18 kg demonstrated better 10-y overall graft survival and death-censored graft survival than adult kidney donors with a KDPI of 30%–85%. Using SRTR data from 1995 to 2007, Kayler et al^23^ showed that pediatric kidney donors weighing >35 kg had a similar graft survival as ideal donors; in practical terms, 35 kg is the approximate median weight of an 11-y-old according to the Centers for Disease Control and Prevention^31^ and weight is a poor predictor of kidney length compared with age or height.^32^ In a similar analysis examining pediatric donors and pediatric recipients, Coens et al^33^ published a retrospective study of all pediatric recipients from 1990 to 2020 using data from the Eurotransplant Registry. They concluded that kidneys from donors aged 2 to 36 y had the lowest risk of graft failure beyond 5 y. Although our study focuses on the inaccuracies of KDPI in assessing pediatric donor quality and outcomes, our analysis is congruent with the studies presented earlier.

The strength of our analyses lies in the sample size of the SRTR database. The large sample size allowed for a reduction in unaccounted biases that were potentially encountered in smaller studies. In contrast to data obtained directly from OPTN, the SRTR database is more robust and a composite database that includes OPTN, Social Security Administration, and Centers for Medicare and Medicaid Services data. The most significant limitation is the nature of the SRTR data set, requiring a complex, detailed knowledge of its structure. As in any retrospective analysis, there may be selection bias as to why some patients receive a kidney transplant from an adult donor. Another limitation of our analysis is the relative short follow-up period of 5 y after transplant. In addition, our final multivariate Cox model does not include several confounders, such as donor race, induction agents, DCD status, or cold ischemic time; this was due to the lack of statistical significance in this sample population and not due to the lack of clinical relevance. KDPI is a calculated index based on donor characteristics from the previous year; therefore, it changes each year. The yearly differences in KDPI for the same donor were not accounted for in this analysis. Despite DCD not being adjusted for in the analysis, the highest proportion of DCD allografts was in the 8–<18-y-old cohorts, which had graft survival similar to that of the cohort with KDPI kidneys ≤20%. SRTR is a national database on solid organ transplantation and may not be applicable to other populations outside the United States. Despite SRTR having a nearly 100% report rate for creatinine at 1 y, it has a <1% report rate at all other follow-up dates; therefore, creatinine cannot be reliably included in a repeated-measure model. Similarly, SRTR states the reliability of immunosuppression reporting is 97% at 1 y; therefore, immunosuppression management cannot be reliably accounted for in any SRTR analysis beyond 1 y. SRTR does not report any tacrolimus drug levels that may affect the outcome. Finally, confounders such as nonadherence to medication, differences in center practices, socioeconomic status, and payor coverage are not directly captured in the SRTR data set.

In summary, our analysis supports that pediatric donors aged 8–18 y have a 5-y death-censored graft survival in adult recipients similar to that of the most “ideal adult deceased donor” with a KDPI ≤20%. Our data also support that recipients with a low EPTS score of ≤20% who are traditionally offered a deceased kidney donor with a KDPI ≤20%, such as pediatric or young adult recipients, may benefit from optimizing kidney allocation for these pediatric donors. In addition, our analysis supports the conclusion that pediatric recipients would likely benefit from accepting pediatric donor kidneys over adult kidney donors with a KDPI >20%. Finally, this analysis highlights the need for an alternative scoring system to KDPI for pediatric donors that more accurately predicts graft outcome.
